# The preoperative albumin-to-carcinoembryonic antigen ratio (ACR) predicts prognosis and facilitates risk stratification in gastric cancer: a retrospective cohort study

**DOI:** 10.3389/fnut.2026.1789564

**Published:** 2026-04-10

**Authors:** Chenshan Yuan, Weigang Wang

**Affiliations:** 1 Department of Clinical Nutrition, Shanxi Province Cancer Hospital, Taiyuan, Shanxi, China; 2 Shanxi Hospital Affiliated to Cancer Hospital, Chinese Academy of Medical Sciences, Taiyuan, Shanxi, China; 3 Cancer Hospital Affiliated to Shanxi Medical University, Taiyuan, Shanxi, China; 4 Department of Clinical Laboratory, Shanxi Province Cancer Hospital, Taiyuan, Shanxi, China

**Keywords:** albumin, carcinoembryonic antigen, gastric cancer, nomogram, prognosis, risk stratification

## Abstract

**Aim:**

This study aimed to evaluate the association between the preoperative albumin-to-carcinoembryonic antigen ratio (ACR) and clinicopathological characteristics as well as the association between ACR and prognosis in gastric cancer, so as to provide evidence for improved risk stratification and personalized management.

**Methods:**

Clinicopathological data of gastric cancer patients who underwent radical gastrectomy at Shanxi Province Cancer Hospital between December 2015 and January 2017 were retrospectively reviewed. Following patient follow-up, Cox proportional-hazards regression was used to identify prognostic factors for overall survival (OS) and disease-free survival (DFS). An ACR-incorporated nomogram was developed, and its predictive accuracy was evaluated using the concordance index (C-index) and calibration curves. Its discriminative ability was further compared against that of the conventional TNM staging system.

**Results:**

Among the 1,161 enrolled patients, 192 (16.5%) and 969 (83.5%) were classified into low- and high-ACR groups, respectively. Low ACR was correlated with adverse pathological features and inferior survival outcomes. Both OS and DFS were significantly shorter in the low-ACR group (both *P* < 0.001). Multivariate analysis identified high ACR as an independent protective factor for OS (HR = 0.741, 95% CI: 0.606–0.906; *P* = 0.003) and DFS (HR = 0.809, 95% CI: 0.655–0.998; *P* = 0.048). Time-dependent receiver operating characteristic (ROC) analysis confirmed the good predictive performance of the ACR-based model in both training and validation sets. The nomogram demonstrated superior predictive accuracy (C-index: 0.748 for OS, 0.730 for DFS) compared with the TNM staging system.

**Conclusion:**

Preoperative low ACR is significantly associated with aggressive tumor biology and poor survival in gastric cancer. The ACR-based nomogram serves as a clinically useful tool for prognostic prediction, risk stratification, and the guidance of personalized therapy.

## 1 Introduction

Gastric cancer ranked as the fifth most commonly diagnosed cancer and the fourth leading cause of cancer-related deaths globally in 2020 (1). Its global burden is projected to escalate substantially, with annual new cases expected to increase from 1.09 to 1.63 million and annual deaths from 0.77 to 1.18 million between 2020 and 2040 (2). Epidemiological patterns exhibit striking geographic variability: the highest incidence rates are observed in Eastern Asia, Eastern Europe, and South America, whereas considerably lower rates prevail across much of North America and Africa (3). Notably, China accounts for a disproportionate share of the global disease burden (4). Despite advances in therapeutic strategies, patient prognosis remains unsatisfactory, with significant survival heterogeneity even among individuals within the same TNM stage. This highlights the critical need for novel biomarkers to complement traditional staging systems, thereby enabling more precise risk stratification and personalized clinical management.

Nutritional status is a well-established determinant of cancer progression, treatment response, and survival outcomes (5, 6). Serum albumin (ALB), which reflects body protein reserves and systemic inflammation, serves as a robust indicator of chronic nutritional status. Hypoalbuminemia not only indicates existing malnutrition but also portends a higher risk of clinical deterioration. By compromising immune competence, malnutrition is particularly detrimental in cancer patients and is associated with an increased risk of postoperative complications (7, 8). Conversely, carcinoembryonic antigen (CEA) is a tumor-derived glycoprotein extensively used in the clinical management of gastric cancer. Elevated preoperative CEA levels are consistently associated with advanced tumor stage, greater tumor burden, and poorer prognosis (9–11).

Prognostic assessment in gastric cancer increasingly utilizes combined inflammatory and nutritional indices, such as the C-reactive protein-albumin-lymphocyte index (CALLY index) (12), D-dimer to albumin ratio (DAR) (13), neutrophil-to-lymphocyte ratio (NLR), platelet-to-lymphocyte ratio (PLR), and lymphocyte-to-monocyte ratio (LMR) (14–16). Building on this paradigm of integrating complementary prognostic information, the albumin-to-carcinoembryonic antigen ratio (ACR) has emerged as a promising composite biomarker. Theoretically, the ACR reflects the balance between host’s nutritional-inflammatory status and tumor aggressiveness. Supporting this rationale, accumulating evidence identifies the ACR as a significant predictor of recurrence and overall survival (OS) in rectal cancer (17). However, the prognostic value of preoperative ACR for disease-free survival (DFS) and OS in gastric cancer remains unexplored, and whether it confers incremental prognostic value beyond established clinicopathological factors is unclear.

Therefore, this study aims to enroll patients with newly diagnosed gastric cancer, systematically collect preoperative clinical data, and perform longitudinal follow-up assessments. The primary objectives are to investigate the association between preoperative ACR and clinicopathological characteristics, evaluate its predictive value for DFS and OS, and thereby provide a theoretical foundation for risk stratification and individualized prognostic management in gastric cancer.

## 2 Patients and methods

### 2.1 Study population and ethics

This was a retrospective analysis of patients diagnosed with and treated for gastric cancer at Shanxi Province Cancer Hospital between December 2015 and January 2017.

Inclusion criteria were as follows:

(1) Age ≥ 18 years with pathologically confirmed gastric cancer;
(2) Primary gastric cancer managed with radical gastrectomy;
(3) No prior treatment for gastric cancer, including surgery, chemotherapy, radiotherapy, or other therapeutic interventions.

Exclusion criteria were:

(1) Receipt of neoadjuvant therapy prior to surgery;
(2) Performance of palliative surgical procedures;
(3) Incomplete data on serum ALB or CEA;
(4) History of other malignant diseases.

This study was approved by the Ethics Review Committee of Shanxi Province Cancer Hospital (Approval No. 2021JCII12). Given the retrospective nature of the study, the requirement for informed consent was waived by the ethics committee.

### 2.2 Data collection

Patient baseline characteristics, laboratory findings, and clinicopathological features were retrieved from the electronic medical record system. Collected data included age, presence of hypertension and diabetes, tumor location, histopathological type, TNM stage, and preoperative serum levels of ALB and CEA, among other variables. The ACR was calculated by dividing the serum ALB concentration (g/L) by the CEA level (μg/L).

### 2.3 Follow-up investigation

Follow-up was conducted through telephone interviews, outpatient visits, and inpatient examinations as clinically indicated. The primary endpoints were disease recurrence, distant metastasis, and survival status. OS was defined as the interval from the date of surgery to death from any cause or the last follow-up. DFS was defined as the time from surgery to the first occurrence of recurrence, death from any cause, or the last follow-up, whichever occurred first. The final follow-up for this study was completed in October 2024.

### 2.4 Statistical analyses

Categorical data were presented as counts and percentages, with group comparisons performed using the χ^2^ test or Fisher’s exact test, as appropriate. Missing data were imputed using multiple imputation by chained equations with the mice package in R. Five imputed datasets were generated with five iterations, employing the random forest algorithm for imputation. The “survminer” R package was used to determine optimal cutoff values for continuous variables, which were then categorized. Specifically, the surv_cutpoint function in the “survminer” R package applies the maximally selected rank statistics (Maxstat) to identify the optimal cut-off points for continuous variables in survival analysis. This method selects the cut-off point that maximizes the log-rank statistic between the high and low groups. Survival analysis was performed using the Kaplan–Meier method to estimate survival probabilities, and differences between groups were assessed using the log-rank test. Univariate and multivariate analyses were carried out using Cox proportional hazards regression models to identify independent prognostic factors.

Visualizations were generated as follows: survival curves were plotted with “survival” and “survminer” R package; restricted cubic spline (RCS) plots were created with “plotRCS” R package; and nomograms were constructed with “rms” R package. Model performance was evaluated using time-dependent receiver operating characteristic (ROC) curves and corresponding area under the curve (AUC) values at predefined time points, implemented with the “timeROC” R package. Calibration and decision curves were plotted using the “rms” and “dcurves” packages, respectively. All statistical analyses were conducted in SPSS (version 27.0) and R (version 4.4.0), with a two-sided *p*-value < 0.05 considered statistically significant.

## 3 Results

### 3.1 OS and DFS comparison in gastric cancer patients across ACR groups

Following the predefined inclusion and exclusion criteria, a total of 1,161 patients diagnosed with gastric cancer were enrolled in this study. The study workflow is illustrated in Supplementary Figure S1. Missing data were handled by multiple imputation using the “mice” R package. No statistically significant differences were observed between the variables before and after imputation (Supplementary Table S1).

The ACR exhibited a broad distribution across the cohort, with a median of 21.74 (range: 0.20–1182.50). Using the “survminer” R package, the optimal prognostic cutoff value for ACR was determined to be 7.18. Based on this threshold, patients were stratified into a low ACR group (≤7.18; *n* = 192, 16.5%) and a high ACR group ( > 7.18; *n* = 969, 83.5%).

**FIGURE 1:**
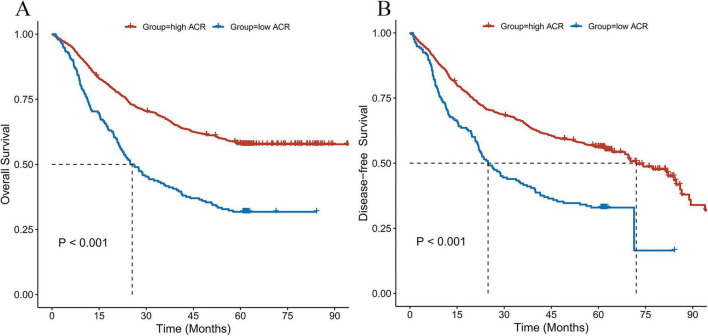
Comparison of OS **(A)** and DFS **(B)** by Kaplan–Meier analysis between high- and low-ACR groups in gastric cancer patients.

Patients in the low ACR group demonstrated significantly worse OS compared with those in the high ACR group (*P* < 0.001; Figure 1A). The 1-, 3-, and 5-year OS rates were 72.4, 42.2, and 31.8% for the low ACR group, compared to 87.1, 67.8, and 57.9% for the high ACR group (Supplementary Table S2). A similar pattern was observed for DFS, with the low ACR group again exhibiting inferior outcomes (*P* < 0.001; Figure 1B, Supplementary Table S2).

**FIGURE 2:**
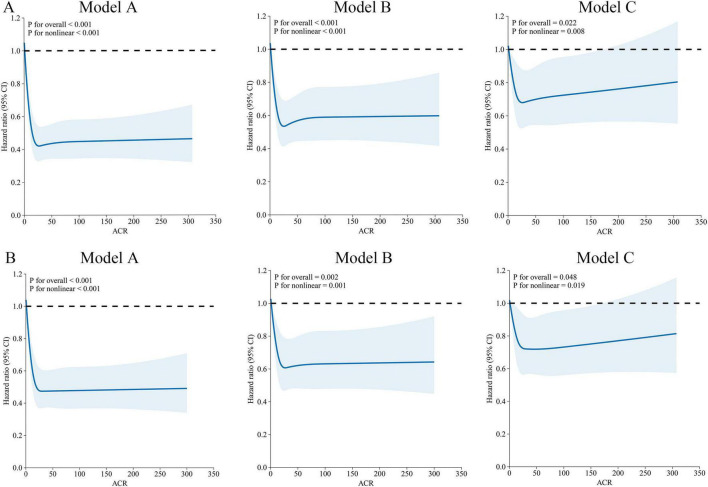
Impact of ACR on OS **(A)** and DFS **(B)** in gastric cancer. Model A (unadjusted): No covariates were adjusted for. Model B (partially adjusted): Adjusted for gender, age, tumor location, histological type, neural invasion, and vascular invasion. Model C (fully adjusted): Additionally adjusted for hypertension, diabetes, smoking, alcohol, T stage, N stage, and M stage.

RCS regression revealed a non-linear, L-shaped relationship between preoperative ACR levels and the risks of both OS and DFS. This pattern indicates that higher ACR levels are associated with a progressively reduced risk of gastric cancer-related mortality and recurrence. Notably, this inverse association remained statistically significant after adjustment for multiple potential confounding factors (Figures 2A,B).

### 3.2 Association of ACR with clinicopathological characteristics

Significant differences in clinicopathological characteristics were observed between the low and high ACR groups, including age, tumor location, vascular invasion, neural invasion, and T, N, M, and TNM stages (all *P* < 0.05; Table 1). These findings suggest that lower ACR levels are associated with more aggressive tumor biology and poorer clinical profiles. Furthermore, several laboratory parameters differed significantly between groups, including neutrophil and monocyte counts, D-dimer levels, tumor markers (CA199, CA242, CA724, CA50), NLR, and PLR (all *P* < 0.05; Supplementary Table S3).

**TABLE 1:** Correlation between ACR and clinicopathological features in gastric cancer patients (*n*, %).

Characteristics	Low ACR (*n* = 192)	High ACR (*n* = 969)	χ^2^	*P*
Gender			1.249	0.264
Male	156 (81.3)	752 (77.6)		
Female	36 (18.7)	217 (22.4)		
Age			4.956	0.026
≤60 year	81 (42.2)	494 (51.0)		
>60 year	111 (57.8)	475 (49.0)		
Smoking			1.771	0.183
Yes	117 (60.9)	540 (55.7)		
No	75 (39.1)	429 (44.3)		
Alcohol			0.021	0.886
Yes	70 (36.5)	348 (35.9)		
No	122 (63.5)	621 (64.1)		
Tumor location			7.893	0.019
Upper third	125 (65.1)	540 (55.7)		
Middle third	22 (11.5)	185 (19.1)		
Lower third	45 (23.4)	244 (25.2)		
Histological type			2.579	0.108
Adenocarcinoma	182 (94.8)	885 (91.3)		
Non-adenocarcinoma	10 (5.2)	84 (8.7)		
Operation method			0.765	0.382
Open	135 (70.3)	650 (67.1)		
Laparoscopy	57 (29.7)	319 (32.9)		
Type of resection			1.609	0.447
Total gastrectomy	87 (45.3)	472 (48.7)		
Proximal subtotal gastrectomy	55 (28.6)	236 (24.4)		
Distal subtotal gastrectomy	50 (26.0)	261 (26.9)		
Vascular invasion			20.145	< 0.001
Yes	123 (64.1)	449 (46.3)		
No	69 (35.9)	520 (53.7)		
Neural invasion			6.989	0.008
Yes	109 (56.8)	449 (46.3)		
No	83 (43.2)	520 (53.7)		
T stage			33.195	< 0.001
T1	1 (0.5)	145 (15.0)		
T2	8 (4.2)	82 (8.5)		
T3	131 (68.2)	496 (51.2)		
T4	52 (27.1)	246 (25.4)		
N stage			72.174	< 0.001
N0	12 (6.3)	310 (32.0)		
N1	19 (9.9)	155 (16.0)		
N2	55 (28.6)	196 (20.2)		
N3	106 (55.2)	308 (31.8)		
M stage			13.565	< 0.001
M0	173 (90.1)	933 (96.3)		
M1	19 (9.9)	36 (3.7)		
TNM stage			65.045	< 0.001
I	4 (2.1)	176 (18.2)		
II	25 (13.0)	247 (25.5)		
III	144 (75.0)	511 (52.7)		
IV	19 (9.9)	35 (3.6)		

**FIGURE 3:**
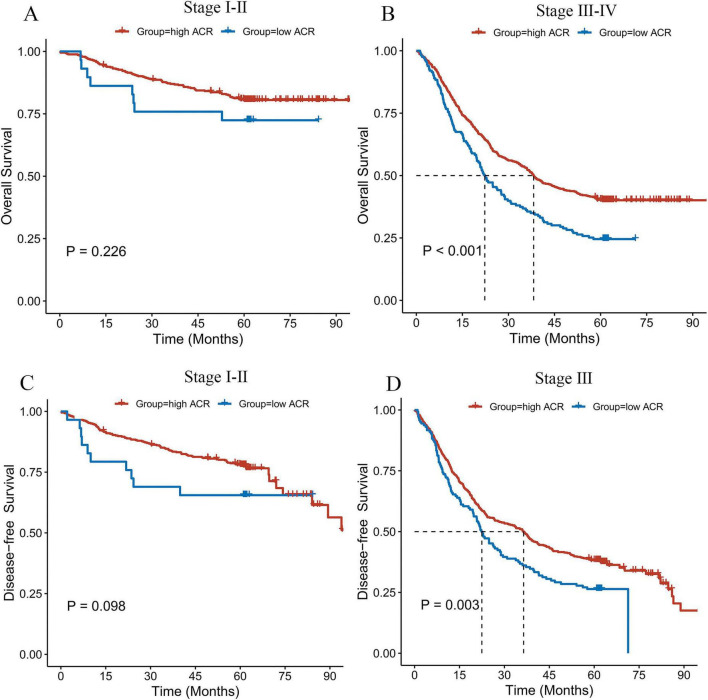
Stratified analysis of the association between ACR and survival outcomes by disease stage. **(A)** OS in early-stage patients. **(B)** OS in advanced-stage patients. **(C)** DFS in early-stage patients. **(D)** DFS in advanced-stage patients.

A significant interaction was found between ACR levels and TNM stage with respect to survival. Among patients with early-stage (I–II) disease, ACR level showed no significant association with OS and DFS (Figures 3A,C). Conversely, in patients with advanced-stage (III–IV) disease, a high ACR was significantly associated with longer OS and DFS (all *P* < 0.05; Figures 3B,D). The prognostic value of ACR was consistent across most T, N, and M substages: low ACR was associated with significantly poorer OS in all subgroups except T1–T2; with this association observed in

T3–T4, all N stages, and M stages (Supplementary Figures S2A–F). A consistent association between low ACR and reduced DFS was also observed across these subgroups (Supplementary Figures S3A–E).

Multivariate subgroup analyses, adjusting for potential confounders, consistently identified ACR as an independent predictor of OS and DFS in the majority of subgroups (Supplementary Figures S4A,B). Sankey diagrams visually underscored the prognostic divergence: the low ACR cohort was characterized by a higher proportion of stage III–IV disease (84.9%, 163/192) and mortality events (68.2%, 131/192), whereas the high ACR group contained fewer advanced-stage patients (56.3%, 546/969) and was associated with more favorable outcomes (42.3%, 410/969) (Supplementary Figure S5A). The association between low ACR and an increased risk of recurrence followed a nearly identical pattern (Supplementary Figure S5B).

Collectively, these results indicate that low ACR is not only correlated with aggressive tumor features but also serves as an independent predictor of adverse survival outcomes in gastric cancer.

### 3.3 Univariate and multivariate analyses of OS and DFS

Multivariate Cox regression analysis identified the following as independent prognostic factors for OS: age (HR = 1.339, 95% CI: 1.129–1.588, *P* = 0.001), vascular invasion (HR = 1.700, 95% CI: 1.390–2.078, *P* < 0.001), neural invasion (HR = 1.333, 95% CI: 1.101–1.614, *P* = 0.003), T stage (HR = 1.917, 95% CI: 1.325–2.773, *P* = 0.001), N stage (HR = 2.858, 95% CI: 2.256–3.620, *P* < 0.001), M stage (HR = 2.146, 95% CI: 1.579–2.916, *P* < 0.001), and ACR level (HR = 0.741, 95% CI: 0.606–0.906, *P* = 0.003) (Table 2). Similarly, ACR level remained an independent prognostic factor for DFS (HR = 0.809, 95% CI: 0.655–0.998, *P* = 0.048) (Table 3).

**TABLE 2:** Univariate and multivariate analysis of OS in patients with gastric cancer.

Characteristics	Univariate analyses		Multivariate analysis	
	HR (95% CI)	*P*	HR (95% CI)	*P*
Gender (male vs. female)	1.001 (0.816–1.228)	0.990		
Age ( > 60 year vs. ≤ 60 year)	1.330 (1.123–1.576)	0.001	1.339 (1.129–1.588)	0.001
Smoking (yes vs. no)	0.895 (0.756–1.060)	0.200		
Alcohol (yes vs. no)	0.907 (0.760–1.083)	0.281		
Hypertension (yes vs. no)	1.099 (0.893–1.353)	0.374		
Diabetes (yes vs. no)	1.163 (0.862–1.569)	0.322		
Tumor location				
Upper third	Ref			
Middle third	1.142 (0.914–1.426)	0.243		
Lower third	0.665 (0.778–1.174)	0.665		
Operation method (laparoscopy vs. open)	1.002 (0.837–1.199)	0.985		
Type of resection				
Total gastrectomy	Ref.			
Proximal subtotal gastrectomy	0.928 (0.752–1.145)	0.488		
Distal subtotal gastrectomy	0.994 (0.813–1.217)	0.955		
Vascular invasion (yes vs. no)	3.258 (2.713–3.912)	< 0.001	1.700 (1.390–2.078)	< 0.001
Neural invasion (yes vs. no)	2.298 (1.929–2.736)	< 0.001	1.333 (1.101–1.614)	0.003
Histological type (adenocarcinoma vs. non-adenocarcinoma)	1.024 (0.749–1.398)	0.883		
T stage (T3, T4 vs. T1, T2)	4.838 (3.449–6.786)	< 0.001	1.917 (1.325–2.773)	0.001
N stage (N3, N2 vs. N1, N0)	4.752 (3.827–5.901)	< 0.001	2.858 (2.256–3.620)	< 0.001
Mstage (M1 vs. M0)	3.404 (2.512–4.613)	< 0.001	2.146 (1.579–2.916)	< 0.001
ACR (high vs. low)	0.473 (0.388–0.576)	< 0.001	0.741 (0.606–0.906)	0.003

**TABLE 3:** Univariate and multivariate analysis of DFS in patients with gastric cancer.

Characteristics	Univariate analyses		Multivariate analysis	
	HR (95% CI)	*P*	HR (95% CI)	*P*
Gender (male vs. female)	1.032 (0.841–1.266)	0.762		
Age (>60 year vs. ≤ 60 year)	1.243 (1.050–1.471)	0.011	1.219 (1.029–1.445)	0.022
Smoking (yes vs. no)	0.969 (0.818–1.148)	0.718		
Alcohol (yes vs. no)	0.978 (0.821–1.165)	0.801		
Hypertension (yes vs. no)	1.097 (0.891–1.351)	0.384		
Diabetes (yes vs. no)	1.204 (0.895–1.619)	0.220		
Tumor location				
Upper third	Ref			
Middle third	1.058 (0.846–1.323)	0.623		
Lower third	0.953 (0.777–1.170)	0.647		
Operation method (laparoscopy vs. open)	0.983 (0.821–1.177)	0.850		
Type of resection				
Total gastrectomy	Ref			
Proximal subtotal gastrectomy	0.934 (0.758–1.151)	0.521		
Distal subtotal gastrectomy	0.989 (0.808–1.211)	0.916		
Vascular invasion (yes vs. no)	2.996 (2.507–3.579)	< 0.001	1.667 (1.372–2.027)	< 0.001
Neural invasion (yes vs. no)	2.261 (1.902–2.689)	< 0.001	1.379 (1.141–1.665)	0.001
Histological type (adenocarcinoma vs. non-adenocarcinoma)	1.070 (0.787–1.457)	0.665		
T stage (T3, T4 vs. T1, T2)	4.361 (3.171–5.998)	< 0.001	1.858 (1.308–2.638)	0.001
N stage (N3, N2 vs. N1, N0)	4.028 (3.294–4.925)	< 0.001	2.641 (2.123–3.286)	< 0.001
ACR (high vs. low)	0.515 (0.419–0.633)	< 0.001	0.809 (0.655–0.998)	0.048

### 3.4 Predictive performance of the ACR-incorporated prognostic model

A Cox regression model was constructed using variables identified as significant in the multivariate analysis. The cohort was randomly split into training (*n* = 813) and validation (*n* = 348) sets at a 7:3 ratio. Baseline characteristics were well balanced between the two sets (Supplementary Table S4).

**FIGURE 4:**
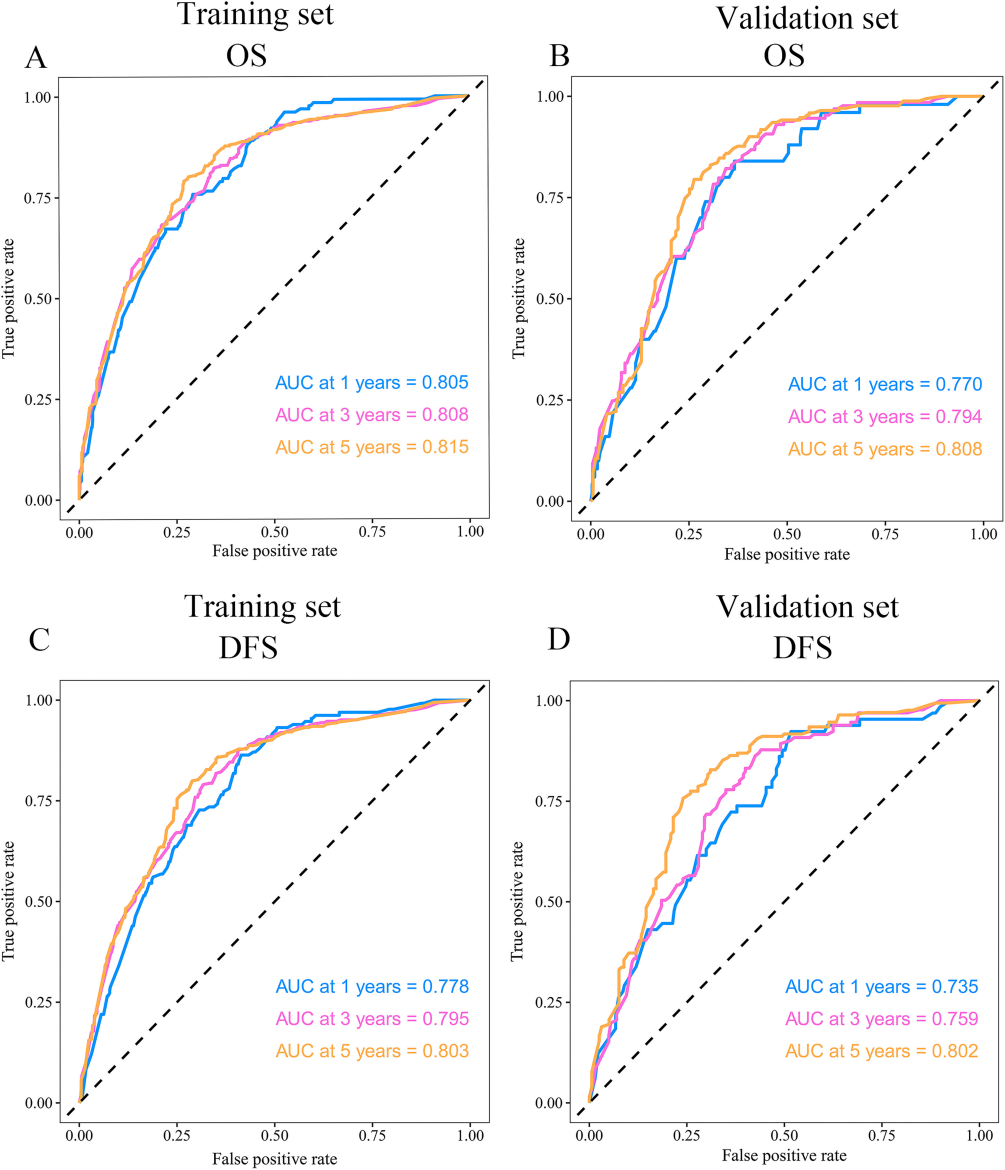
Predictive performance of the ACR-incorporated prognostic model assessed by time-dependent ROC curves. Shown are the curves for OS in the training and validation sets (**A,B**, respectively), and for DFS in the two sets (**C,D**, respectively).

Time-dependent ROC analysis was used to evaluate the model’s predictive accuracy for 1-, 3-, and 5-year OS and DFS. In the training set, the AUC values for OS were 0.805, 0.808, and 0.815, respectively (Figure 4A); corresponding values in the validation set were 0.770, 0.794, and 0.808 (Figure 4B). For DFS, the training set achieved AUCs of 0.778, 0.795, and 0.803 (Figure 4C), while the validation set showed AUCs of 0.735, 0.759, and 0.802 (Figure 4D). Calibration plots demonstrated good agreement between predicted and observed probabilities for both OS (Supplementary Figures S6A,B) and DFS (Supplementary Figures S7A,B), confirming the model’s robustness and clinical applicability. Decision curve analysis showed that, for both OS (Supplementary Figures S8A,B) and DFS (Supplementary Figures S9A,B), the ACR-integrated nomogram consistently outperformed TNM staging in both the training and validation sets.

### 3.5 Development of an ACR-incorporated nomogram

**FIGURE 5:**
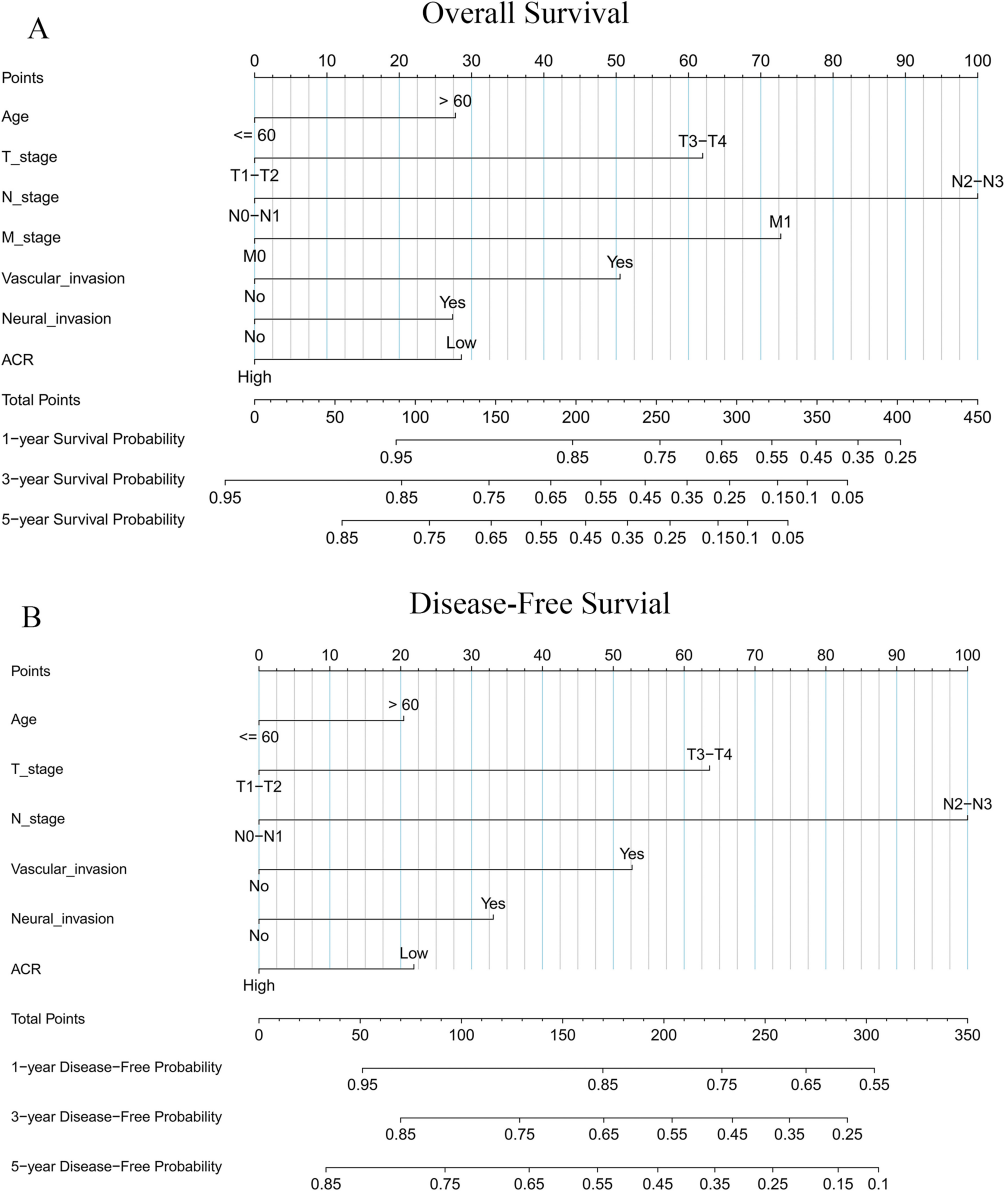
Prognostic nomograms derived from the ACR-incorporated model for predicting OS **(A)** and DFS **(B)**.

Based on the independent prognostic factors identified, two nomograms were constructed to predict OS (Figure 5A) and DFS (Figure 5B) in patients with gastric cancer. The models demonstrated good discriminative ability, with C-indices of 0.748 for OS and 0.731 for DFS.

Using the nomogram scoring system, total points were calculated for each patient. Optimal cutoff values of 142 for OS and 137 for DFS—determined via the survminer package—were employed to categorize patients into high-risk and low-risk groups. Kaplan–Meier analysis confirmed significantly poorer OS (Supplementary Figure S10A; *P* < 0.001) and DFS (Supplementary Figure S10B; *P* < 0.001) in the high-risk group, thus validating the nomogram’s capacity to identify patients with poor prognosis.

### 3.6 Comparison of the ACR-incorporated nomogram with the TNM staging system

**FIGURE 6:**
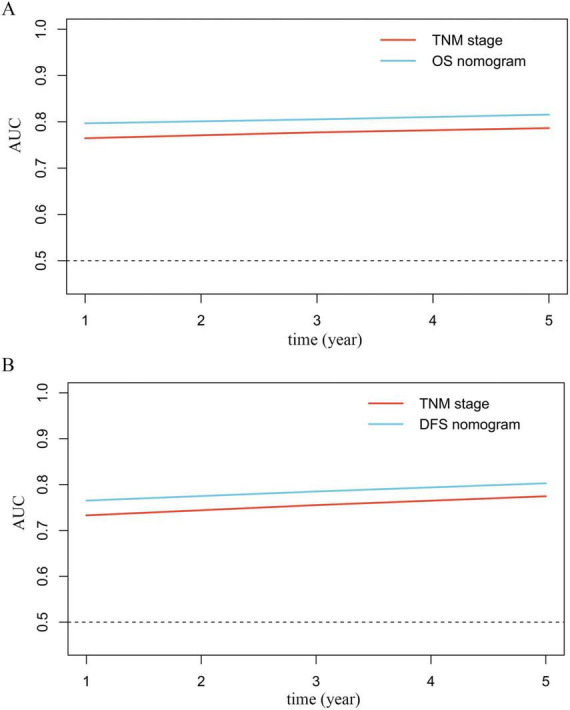
Time-dependent ROC curves comparing the prognostic performance of the ACR-incorporated nomogram and the conventional TNM staging system for OS **(A)** and DFS **(B)**.

Time-dependent ROC analysis indicated that the nomogram provided good predictive accuracy for both OS (Figure 6A) and DFS (Figure 6B) compared with the conventional TNM staging system. This superiority was quantitatively confirmed by significant improvements in the C-index, net reclassification improvement (NRI), and integrated discrimination improvement (IDI). For OS, the nomogram yielded a 2.4% increase in the C-index, a 25.6% improvement in NRI, and a 44.5% improvement in IDI (all *P* < 0.05; Table 4). Similarly, for DFS, corresponding improvements were 2.1% in the C-index, 25.0% in NRI, and 44.6% in IDI (all *P* < 0.05; Table 4).

**TABLE 4:** Comparison of discriminatory ability between nomograms and TNM stage for gastric cancer.

Model	C index			NRI		IDI	
	Value	Difference	*P*	Difference	*P*	Difference	*P*
OS							
TNM stage	0.724	Ref.		Ref.		Ref.	
OS nomogram	0.748	0.024 (0.015–0.034)	< 0.001	0.256 (0.080–0.399)	0.002	0.045 (0.020–0.083)	< 0.001
DFS							
TNM stage	0.707	Ref.		Ref.		Ref.	
DFS nomogram	0.730	0.021 (0.015–0.036)	< 0.001	0.250 (0.092–0.392)	0.002	0.046 (0.018–0.085)	< 0.001

## 4 Discussion

Despite advances in the diagnosis and treatment of gastric cancer, long-term survival outcomes remain suboptimal, underscoring the need for improved prognostic tools. In this study, we identified low preoperative ACR as a factor correlated with adverse clinicopathological features and inferior survival. We subsequently developed and validated an ACR-based prognostic model that demonstrates robust predictive performance and effectively stratifies patient risk, offering a practical tool to aid in personalized treatment planning.

Malnutrition is highly prevalent among patients with cancer, often as a consequence of chronic disease. Those with gastrointestinal malignancies are at particularly high risk, as the functional integrity of the gastrointestinal tract is a key determinant of nutritional status. Serum ALB, synthesized in the liver, is an established biomarker for assessing systemic nutrition status. Substantial evidence links hypoalbuminemia to poorer prognosis and disease progression across various malignancies, including colon (18), colorectal (19), pancreatic (20), non-small cell lung (21), ovarian (22), and gastric cancers (23). Several albumin-derived indices have shown prognostic value in gastric cancer. For instance, the CALLY index has been incorporated into a nomogram alongside TNM stage and body mass index, demonstrating superior discriminatory ability compared with the TNM system alone (24). Studies have demonstrated that the postoperative ratio of C-reactive protein to albumin (HR = 2.03) can serve as an independent prognostic factor for gastric cancer (25). The albumin-to-globulin ratio (AGR) is another prognostic biomarker: a low AGR independently predicts worse OS (HR = 1.380) and progression-free survival (PFS: HR = 1.514) in patients with metastatic gastric cancer (26). A meta-analysis confirms this, reporting that a low AGR is significantly associated with poorer OS (HR = 1.531) and DFS/PFS (HR = 2.008) in gastric cancer (27). Similarly, the preoperative DAR can predict survival and recurrence in patients with gastric cancer (13).

CEA is a well-established biomarker in gastric cancer, with elevated levels closely associated with metastasis and recurrence. Studies indicate that elevated preoperative CEA increases the risk of early postoperative recurrence, particularly within the first 2 years (10). Furthermore, serial CEA measurements can help evaluate treatment response— serial CEA predicting pathological complete response after neoadjuvant chemotherapy in advanced gastric cancer (28). Integrating CEA with other markers, such as the NLR in the CNLR score, has yielded prognostic nomograms, although the C-index (0.704) (29) is lower than that observed in our study (0.748). Additionally, the combination of NLR and CEA may be used to assess the efficacy of postoperative adjuvant chemotherapy in gastric cancer (30), highlighting its potential clinical utility. Other combinations, such as CEA with hemoglobin (31) or the fibrinogen-to-albumin ratio (FAR) (32), also provide practical prognostic information.

Combining readily available biomarkers into composite indices represents a promising strategy in oncology, minimizing additional costs while offering clinicians more comprehensive prognostic tools. A substantial body of evidence underscores the critical role of the tumor microenvironment in tumor initiation and progression. Within this milieu, tumor cells reside within a complex stromal network composed of vascular cells, immune cells, fibroblasts, and other cellular components (33). Consequently, nutritional status, inflammatory cues, and tumor burden collectively regulate the plasticity of both tumor cells and surrounding stromal cells, profoundly influencing their phenotypic and functional evolution (24). The ACR, which integrates ALB and CEA, encapsulates information on nutritional status, systemic inflammation, and tumor burden, thereby offering considerable prognostic utility. Prior studies support this concept; for example, a preoperative ALB-CEA score has been proposed as a prognostic biomarker in gastric cancer (8), and a low ACR has been independently associated with aggressive tumor behaviours and poor survival in rectal cancer (17). Our findings confirm that a low preoperative ACR is significantly associated with unfavourable clinicopathological characteristics and worse prognosis in patients with gastric cancer. We therefore recommend incorporating preoperative ACR assessment into routine clinical practice. Patients with a low ACR warrant closer surveillance and intensified postoperative follow-up to enable more precise risk-stratified management. The ACR-based prognostic model developed in this study demonstrated significantly higher predictive accuracy for both OS and DFS than the conventional TNM staging system (C-index: OS 0.748 vs. 0.724; DFS 0.730 vs. 0.707), supporting its role as a reliable supplementary tool for refined risk stratification. The nomogram performed slightly better for OS than for DFS, a discrepancy possibly attributable to the fact that the optimal ACR cutoff was derived based on OS within this cohort. Importantly, both CEA and ALB are routine, cost-effective blood tests, ensuring the clinical feasibility and broad applicability of the ACR.

Several limitations of this study should be acknowledged. Its retrospective, single-center design introduces potential selection bias. Moreover, given that ALB and CEA levels fluctuate dynamically in response to treatment and disease progression—thereby impacting ACR variability—relying solely on static preoperative ACR measurements may not fully capture its prognostic value. To address this limitation, our subsequent studies will focus on dynamically tracking ACR trajectories, which will allow us to clarify the impact of longitudinal ACR patterns on the prognosis of gastric cancer patients. Most importantly, the absence of an external validation cohort necessitates cautious interpretation of our findings. Future multi-center prospective studies are essential for verification.

## 5 Conclusion

This study identifies the preoperative ACR as a significant prognostic marker in gastric cancer, with a low ACR indicating adverse clinicopathological features and inferior survival outcomes. The ACR-based nomogram demonstrates good discriminative ability compared with the traditional TNM staging system, thereby enabling individualized prediction of recurrence risk. This model may facilitate the tailoring therapeutic strategies and support more personalized patient management.

## Data Availability

The original contributions presented in the study are included in the article/Supplementary material, further inquiries can be directed to the corresponding author.
