# Supplementary material for: The preoperative albumin-to-carcinoembryonic antigen ratio (ACR) predicts prognosis and facilitates risk stratification in gastric cancer: a retrospective cohort study
Source: Front Nutr. 2026 Apr 10;13:1789564. doi: 10.3389/fnut.2026.1789564 (PMC13105995; doi:10.3389/fnut.2026.1789564)
Supplement: Supplementary file 1 [file Table_1.docx]

Supplementary Material

# Supplementary Figures

**Figure S1.** Participant flowchart.


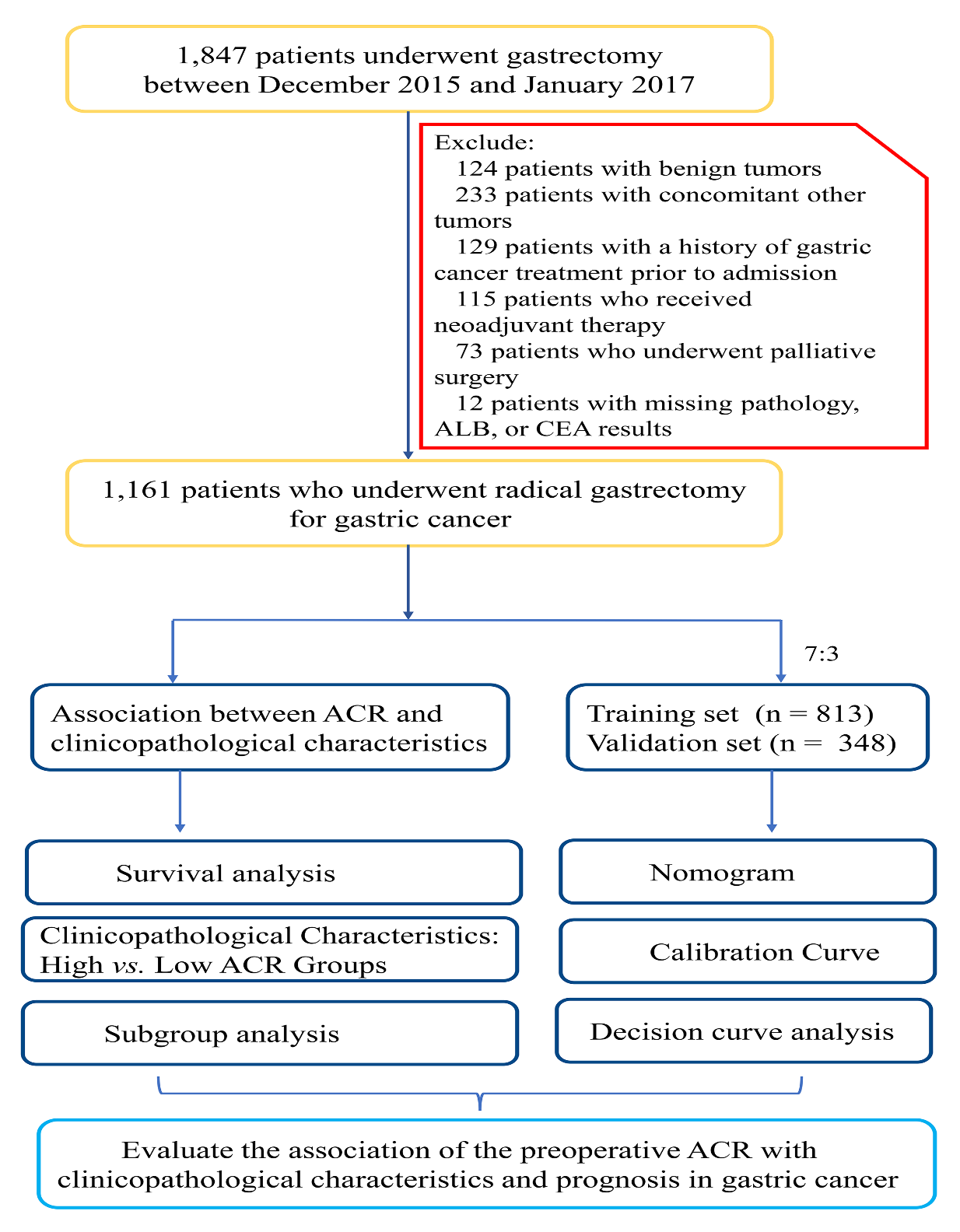


**Figure S2.** Comparison of OS according to ACR levels, stratified by T stage (A, B), N stage (C, D), and M stage (E, F).


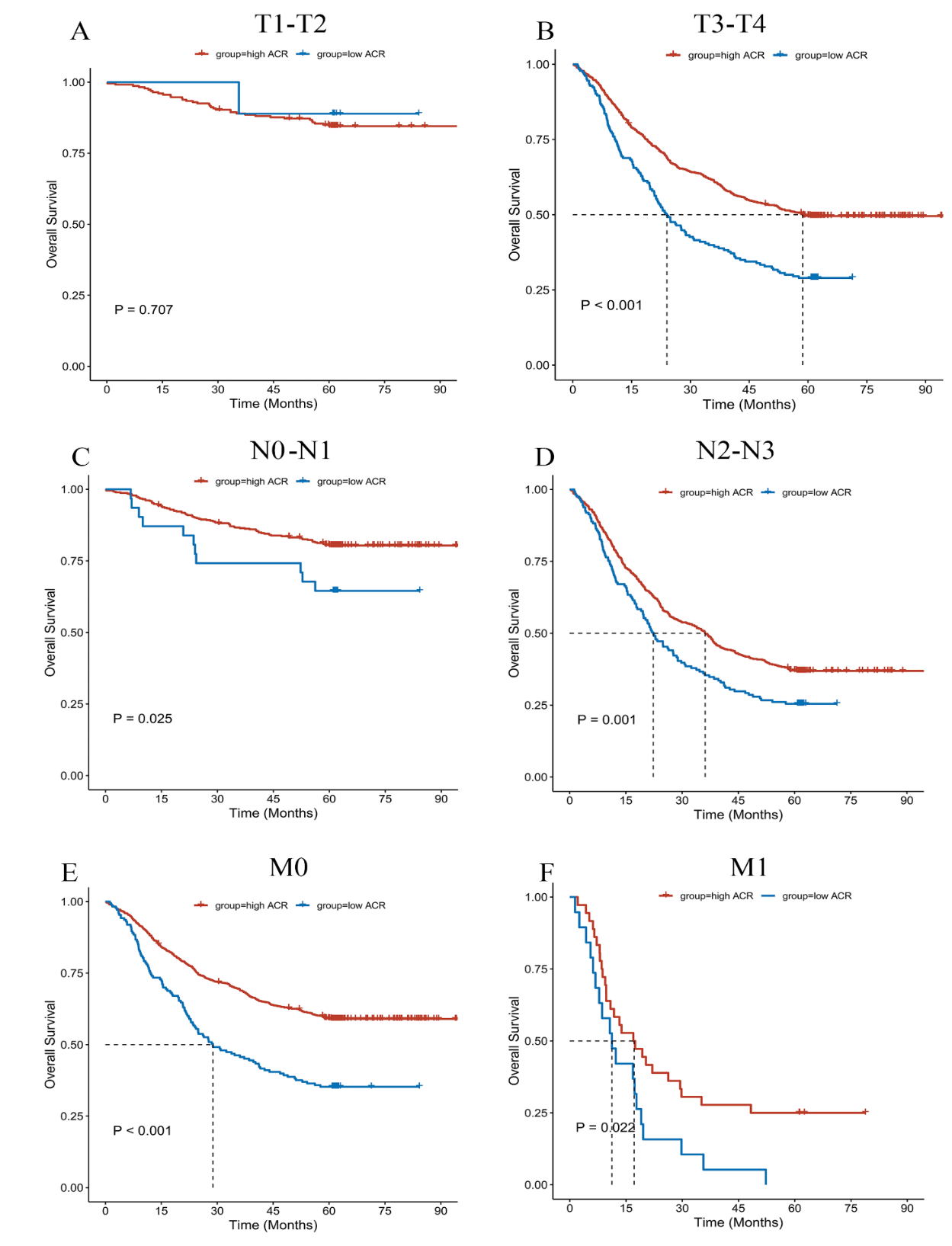


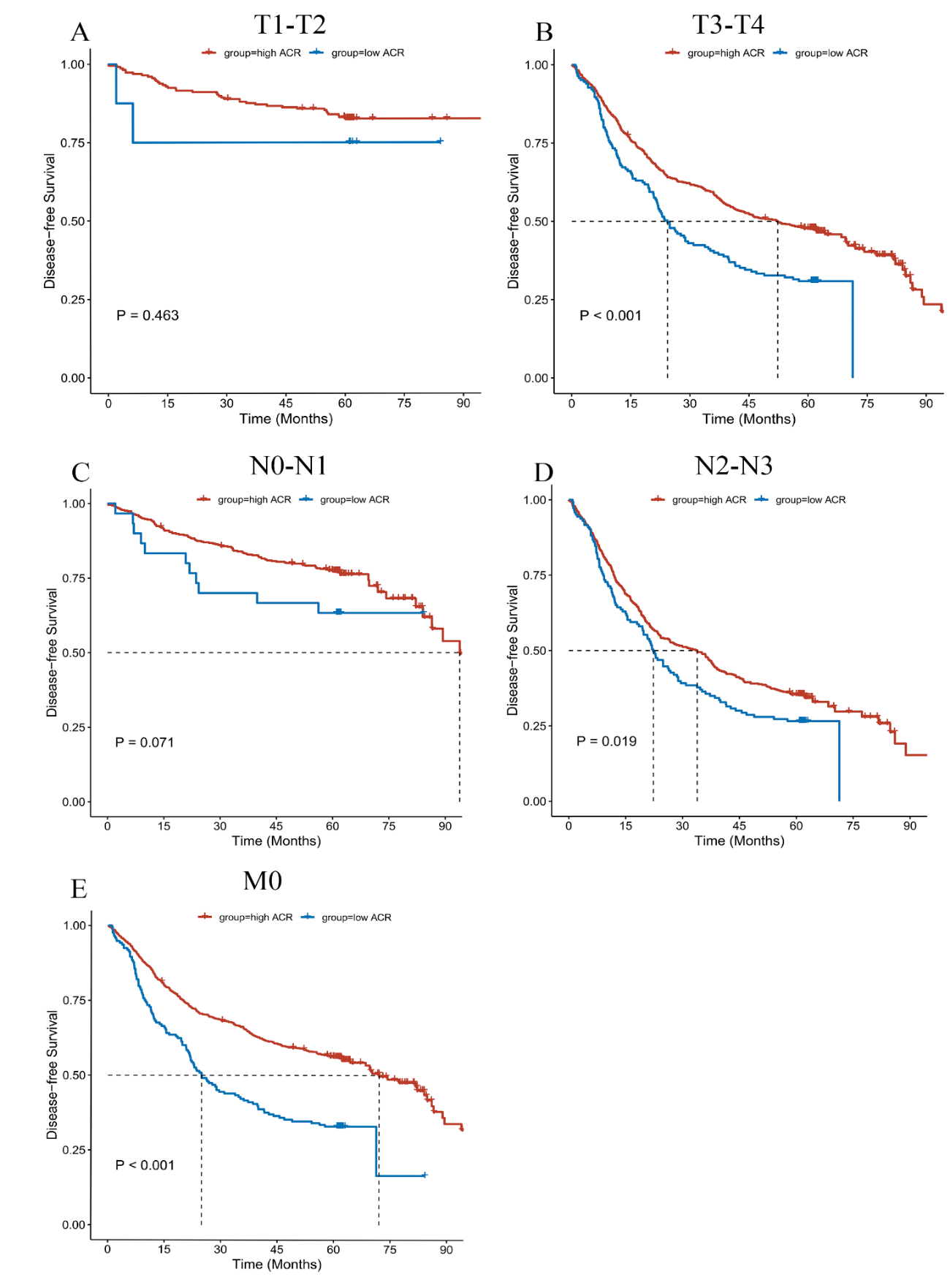
**Figure S3.** Comparison of DFS according to ACR levels, stratified by T stage (A, B), N stage (C, D), and M stage (E).

**Figure S4.** Association of ACR with the hazard risk of OS (A) and DFS (B) across patient subgroups.


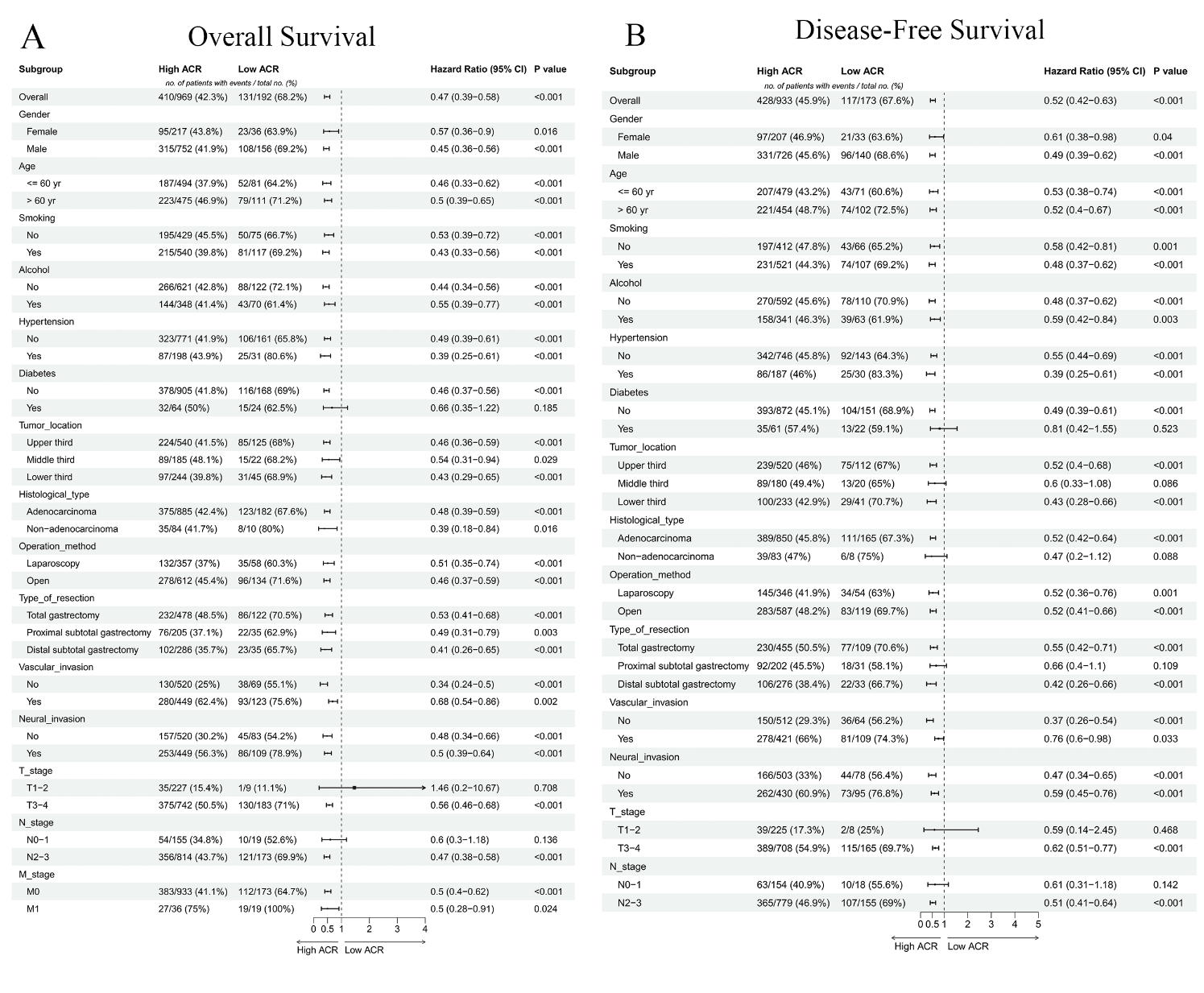


**Figure S5**. Sankey diagram depicting the relationship between ACR levels, TNM stage, and survival outcomes (OS and DFS). (A) OS. (B) DFS.


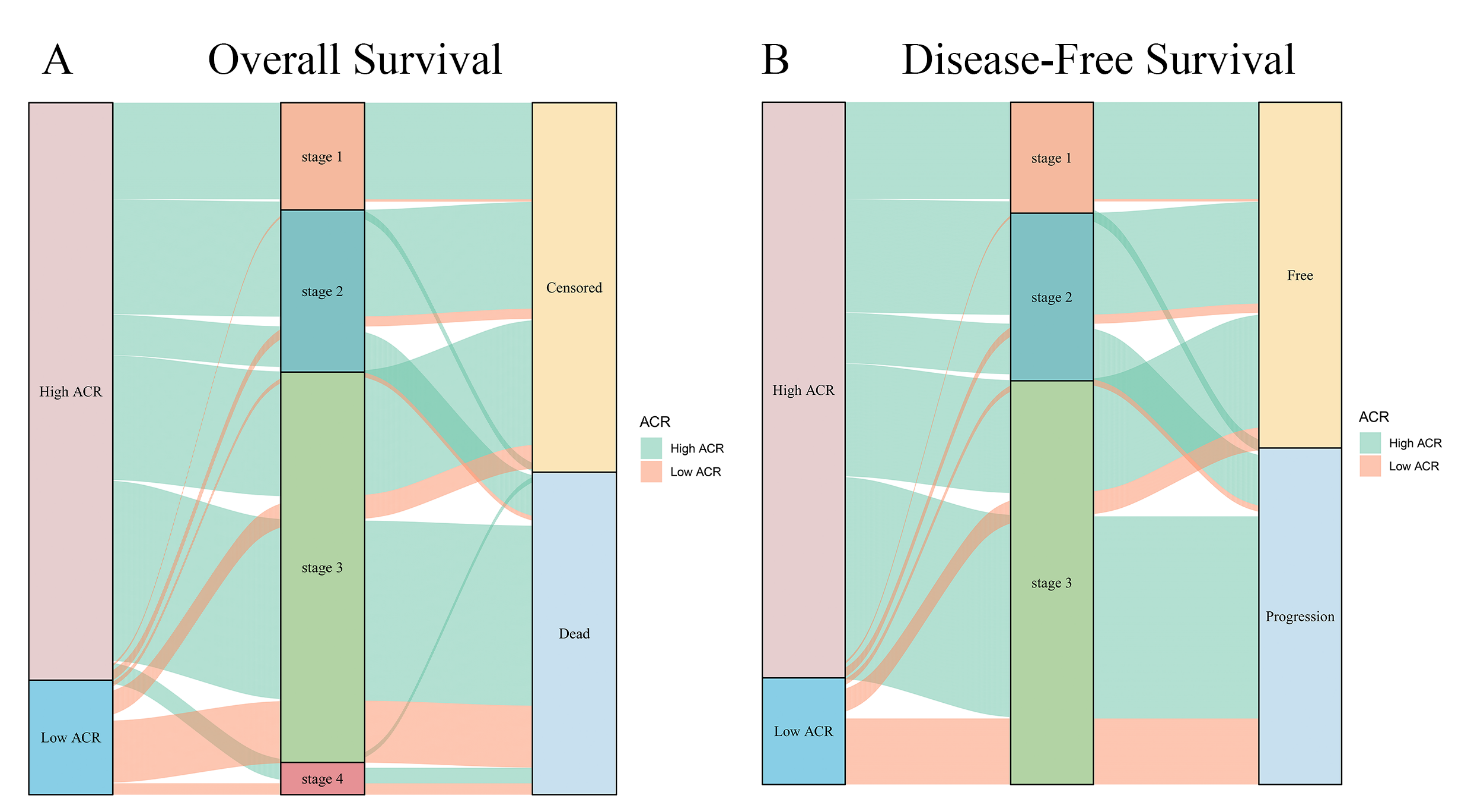


**Figure S6.** Validation of the predictive accuracy of the OS nomograms for 1-,3‑ and 5‑year survival in the training (A) and validation sets (B).


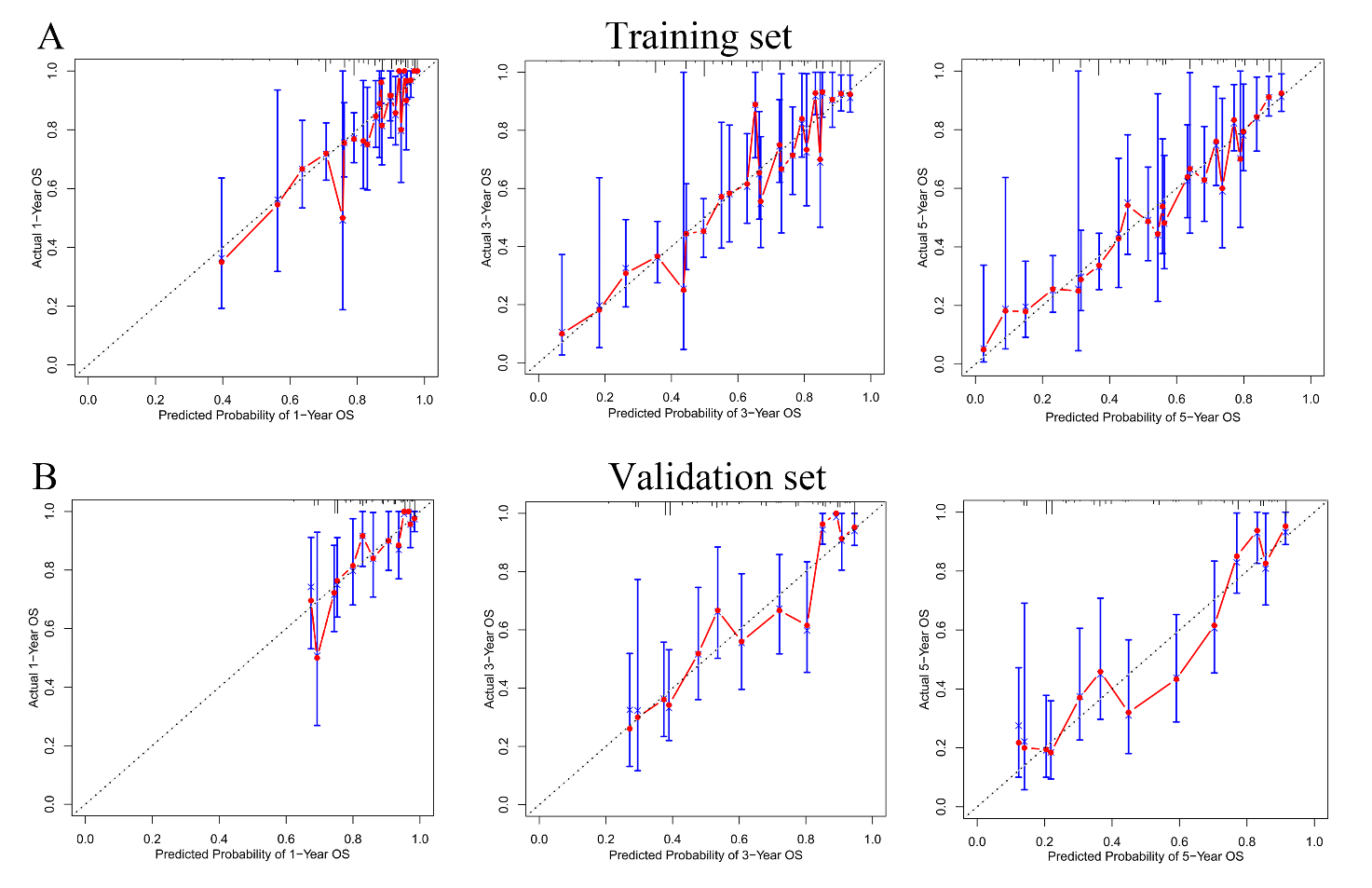


**Figure S7.** Validation of the predictive accuracy of the DFS nomograms for 1-,3‑ and 5‑year survival in the training (A) and validation sets (B).


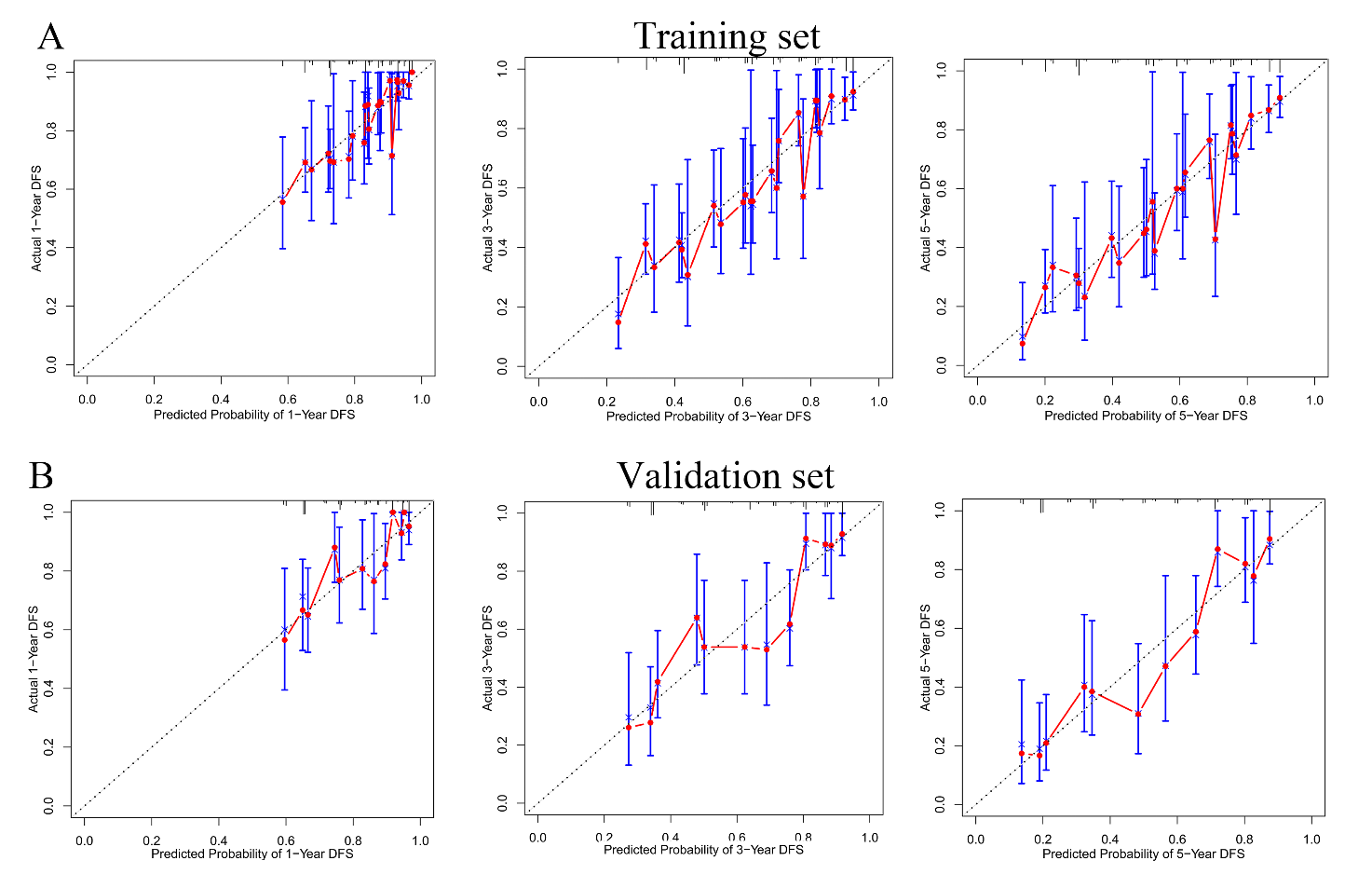


**Figure S8.** Decision curve analysis assessing the clinical utility of the OS nomogram versus the TNM staging system in the training (A) and validation sets (B).


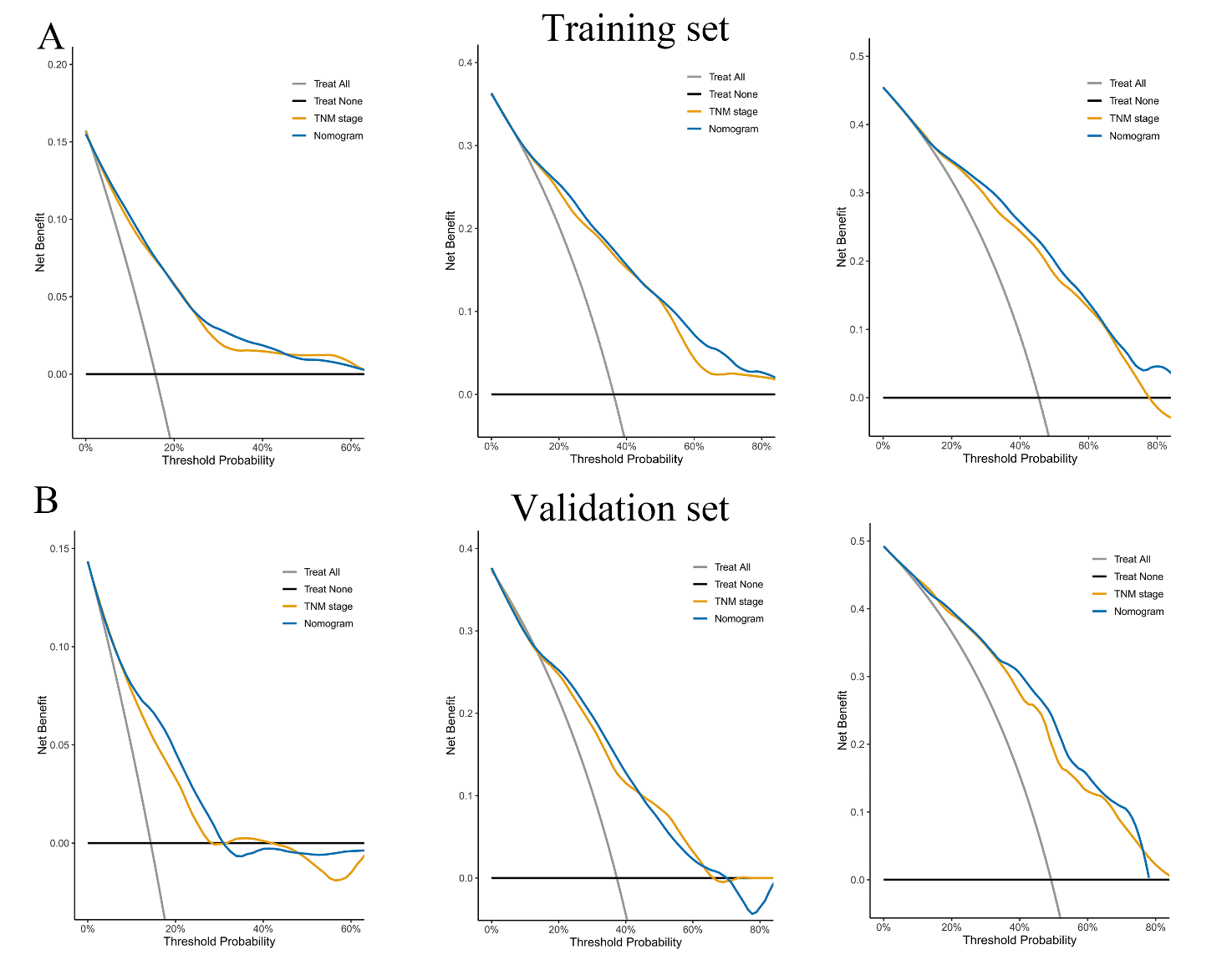


**Figure S9.** Decision curve analysis assessing the clinical utility of the DFS nomogram versus the TNM staging system in the training (A) and validation sets (B).


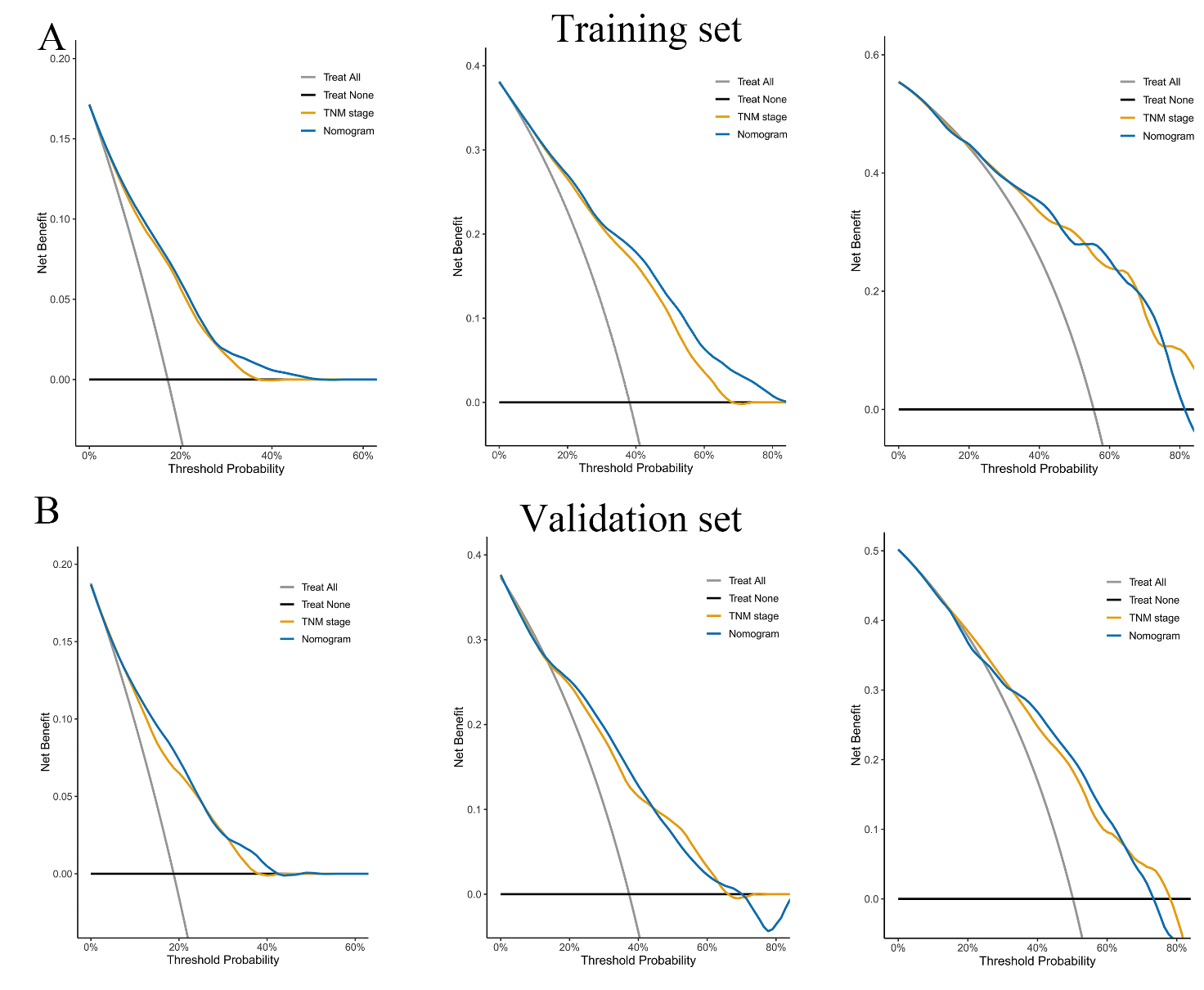


**Figure S10.** Risk stratification by nomogram total points and subsequent survival analysis. (A) OS. (B) DFS.


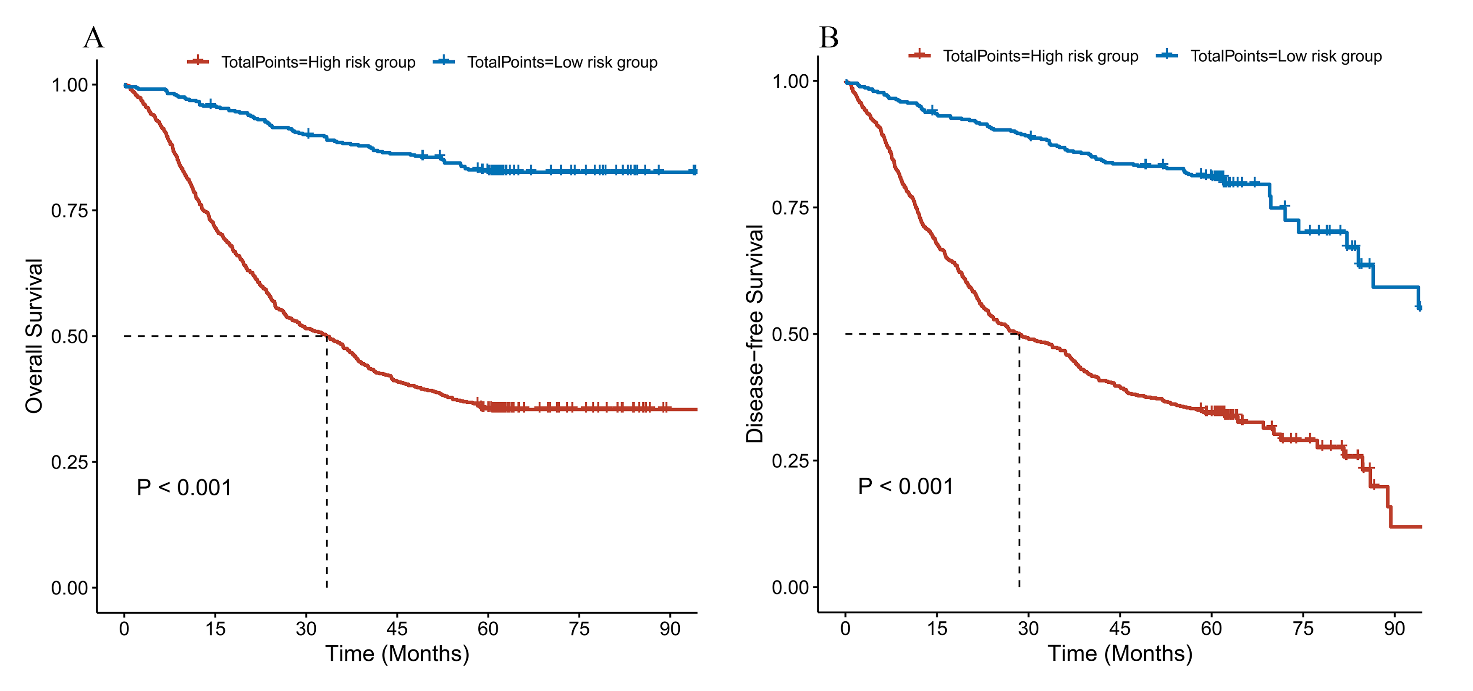


# Supplementary Tables

Table S1 Comparison of data before and after imputation

| Variable | Before imputation | After imputation | Z/χ2 | P |
| --- | --- | --- | --- | --- |
| Gender (n, %) |  |  | - | - |
| Male | 908 (78.2) | - |  |  |
| Female | 253 (21.8) | - |  |  |
| Age at diagnosis (median, IQR), years | 61.00 (13.00) | - | - | - |
| Smoking (n, %) |  |  | 0.017 | 0.897 |
| Yes | 637 (56.3) | 657 (56.6) |  |  |
| No | 494 (43.7) | 504 (43.4) |  |  |
| Alcohol (n, %) |  |  | 0.108 | 0.742 |
| Yes | 382 (35.3) | 418 (36.0) |  |  |
| No | 699 (64.7) | 743 (64.0) |  |  |
| Hypertension (n, %) |  |  | - | - |
| Yes | 229 (19.7) | - |  |  |
| No | 932 (80.3) | - |  |  |
| Diabetes (n, %) |  |  | - | - |
| Yes | 88 (7.6) | - |  |  |
| No | 1073 (92.4) | - |  |  |
| Location (n, %) |  |  | - | - |
| Upper third | 665 (57.3) | - |  |  |
| Middle third | 207 (17.8) | - |  |  |
| Lower third | 289 (24.9) | - |  |  |
| Histological type (n, %) |  |  | - | - |
| Adenocarcinoma | 1067 (91.9) | - |  |  |
| Non-adenocarcinoma | 94 (8.1) | - |  |  |
| Operation method (n, %) |  |  | - | - |
| Open | 785 (67.6) | - |  |  |
| Laparoscopy | 376 (32.4) | - |  |  |
| Type of resection (n, %) |  |  | - | - |
| Total gastrectomy | 559 (48.1) | - |  |  |
| Proximal subtotal gastrectomy | 291 (25.1) | - |  |  |
| Distal subtotal gastrectomy | 311 (26.8) | - |  |  |
| Vascular invasion (n, %) |  |  | 0.253 | 0.615 |
| Yes | 366 (48.1) | 572 (49.3) |  |  |
| No | 395 (51.9) | 589 (50.7) |  |  |
| Neural invasion (n, %) |  |  | 0.116 | 0.733 |
| Yes | 556 (48.8) | 558 (48.1) |  |  |
| No | 584 (51.2) | 603 (51.9) |  |  |
| T stage (n, %) |  |  | 0.321 | 0.956 |
| T1 | 134 (11.8) | 146 (12.6) |  |  |
| T2 | 88 (7.8) | 90 (7.8) |  |  |
| T3 | 619 (54.5) | 627 (54.0) |  |  |
| T4 | 294 (25.9) | 298 (25.7) |  |  |
| N stage (n, %) |  |  | 0.026 | 0.999 |
| N0 | 316 (27.5) | 322 (27.7) |  |  |
| N1 | 174 (15.1) | 174 (15.0) |  |  |
| N2 | 249 (21.6) | 251 (21.6) |  |  |
| N3 | 412 (35.8) | 414 (35.7) |  |  |
| M stage (n, %) |  |  |  |  |
| M0 | 1106 (95.3) |  |  |  |
| M1 | 55 (4.7) |  |  |  |
| Neutrophil (median, IQR), (× 10^9^/L) | 3.62 (1.69) | - | - | - |
| Lymphocyte (median, IQR), (× 10^9^/L) | 1.87 (0.80) | - | - | - |
| Monocyte (median, IQR), (× 10^9^/L) | 0.38 (0.18) | 0.38 (0.17) | -0.265 | 0.791 |
| Platelet (median, IQR), (× 10^9^/L) | 247 (96.00) | - | - | - |
| D-dimer (median, IQR), (mg/L) | 105.00 (107.00) | 106.00 (106.00) | -0.228 | 0.819 |
| ALB (median, IQR), (g/L) | 43.00(5.20) | - | - | - |
| CEA (median, IQR), (μg/L) | 2.00 (2.45) | - | - | - |
| CA199 (median, IQR), (U/mL) | 11.45 (16.21) | - | - | - |
| CA242 (median, IQR), (U/mL) | 4.08 (8.53) | 4.08 (8.53) | -0.027 | 0.978 |
| AFP (median, IQR) (ng/mL) | 2.25 (2.94) | 2.25 (2.95) | -0.024 | 0.981 |
| CA724 (median, IQR) (U/mL) | 2.79 (4.62) | 2.79 (4.60) | -0.019 | 0.985 |
| CA50 (median, IQR), (U/mL) | 1.42 (2.38) | 1.39 (2.33) | -0.374 | 0.708 |
| SCC (median, IQR), (ng/mL) | 0.34 (0.33) | 0.34 (0.33) | -0.140 | 0.889 |
| TPS (median, IQR), (U/L) | 88.82 (84.42) | 88.65 (84.23) | -0.039 | 0.969 |
| CD45+CD3+T cell (median, IQR), (%) | 68.90 (14.40) | 69.00 (14.10) | -0.065 | 0.948 |
| CD3+CD4+ T cell (median, IQR), (%) | 35.90 (12.00) | 36.00 (11.90) | -0.084 | 0.933 |
| CD3+CD8+ T cell (median, IQR), (%) | 27.30 (13.10) | 27.50 (13.10) | -0.050 | 0.960 |
| CD4+CD8+ T cell (median, IQR), (%) | 0.90 (1.00) | 1.00 (1.00) | -0.035 | 0.972 |
| CD3+CD4+ T cell / CD3+CD8+ T cell ratio (median, IQR), | 1.30 (0.95) | 1.29 (0.97) | -0.221 | 0.825 |
| CD45+CD3-CD16+CD56+ T cell (median, IQR), (%) | 19.90 (13.90) | 19.80 (13.80) | -0.168 | 0.867 |
| CD3+CD56+ T cell (median, IQR), (%) | 5.50 (6.30) | 5.60 (6.40) | -0.176 | 0.860 |
| CD45+CD3-CD25^high^CD127^low^ T cell (median, IQR), (%) | 5.90 (2.40) | 5.90 (2.40) | -0.111 | 0.911 |
| CD45+CD3-CD19+ B cell (median, IQR), (%) | 8.40 (5.50) | 8.50 (5.50) | -0.036 | 0.971 |
| SIL2R (median, IQR), (U/ml) | 328.00 (77.00) | 327.00 (76.00) | -0.152 | 0.879 |
| TSGF (median, IQR), (U/ml) | 59.00 (11.00) | 59.00 (11.00) | -0.133 | 0.894 |

Table S2 Comparison of 1-, 3-, and 5-year OS and DFS across different ACR groups

| Variable | Low ACR | High ACR |
| --- | --- | --- |
| OS |  |  |
| 1-year | 72.4% (95% CI: 66.3% - 79.0%) | 87.1% (95% CI: 85.0% - 89.2%) |
| 3-year | 42.2% (95% CI: 35.7% - 49.8%) | 67.8% (95% CI: 64.9% - 70.8%) |
| 5-year | 31.8% (95% CI: 25.8% - 39.1%) | 57.9% (95% CI: 54.9% - 61.1%) |
| DFS |  |  |
| 1-year | 69.9% (95% CI: 63.4% - 77.1%) | 84.5% (95% CI: 82.2% - 86.8%) |
| 3-year | 42.2% (95% CI: 35.4% - 50.2%) | 65.9% (95% CI: 62.9% - 69.0%) |
| 5-year | 32.9% (95% CI: 26.6% - 40.8%) | 56.3% (95% CI: 53.2% - 59.6%) |

Table S3 Correlation between ACR and laboratory findings in patients with gastric cancer (M, IQR)

| Variable | Low ACR  (n = 192) | High ACR  (n = 969) | Z | P |
| --- | --- | --- | --- | --- |
| Neutrophil | 3.82 (2.01) | 3.58 (1.69) | -2.490 | 0.013 |
| Lymphocyte | 1.85 (0.84) | 1.87 (0.79) | -0.351 | 0.725 |
| Monocyte | 0.39 (0.21) | 0.37 (0.18) | -2.214 | 0.027 |
| Platelet | 245.50 (93.50) | 247.00 (96.00) | -0.174 | 0.862 |
| D-dimer | 122.50 (155.00) | 103.00 (99.00) | -3.082 | <0.001 |
| ALB | 41.85 (5.80) | 43.10 (5.00) | -3.661 | <0.001 |
| CEA | 17.79 (36.27) | 1.61 (1.56) | -21.902 | <0.001 |
| CA199 | 13.91 (51.55) | 11.02 (13.93) | -3.332 | 0.001 |
| CA242 | 6.28 (44.23) | 3.70 (7.23) | -4.462 | <0.001 |
| AFP | 2.37 (3.65) | 2.24 (2.77) | -1.565 | 0.118 |
| CA724 | 3.76 (7.72) | 2.64 (4.18) | -4.778 | <0.001 |
| CA50 | 1.60 (6.23) | 1.34 (2.09) | -3.194 | 0.001 |
| SCC | 0.38 (0.47) | 0.34 (0.33) | -1.177 | 0.239 |
| TPS | 92.53 (97.41) | 87.97 (82.00) | -1.664 | 0.096 |
| CD45+CD3+T cell | 68.20 (16.20) | 69.00 (13.70) | -0.410 | 0.682 |
| CD3+CD4+ T cell | 35.75 (11.92) | 36.00 (11.95) | -0.102 | 0.918 |
| CD3+CD8+ T cell | 27.05 (13.20) | 27.50 (13.10) | -0.566 | 0.572 |
| CD4+CD8+ T cell | 0.95 (0.90) | 1.00 (1.10) | -0.352 | 0.725 |
| CD3+CD4+ T cell / CD3+CD8+ T cell ratio | 1.31 (0.99) | 1.29 (0.97) | -0.410 | 0.682 |
| CD45+CD3-CD16+CD56+ T cell | 20.70 (15.30) | 19.70 (13.60) | -0.749 | 0.454 |
| CD3+CD56+ T cell | 5.50 (5.90) | 5.60 (6.40) | -0.064 | 0.949 |
| CD45+CD3-CD25highCD127low T cell | 58.0 (2.30) | 5.90 (2.40) | -0.003 | 0.998 |
| CD45+CD3-CD19+ B cell | 7.96 (5.10) | 8.50 (5.60) | -1.305 | 0.192 |
| SIL2R | 326.00 (74.00) | 328.00 (77.00) | -0.351 | 0.726 |
| TSGF | 59.00 (11.00) | 59.00 (10.00) | -1.523 | 0.128 |
| NLR (Neutrophil-to-Lymphocyte Ratio) | 1.99 (1.19) | 1.90 (1.06) | -2.055 | 0.040 |
| PLR (Platelet-to-Lymphocyte Ratio) | 135.36 (76.87) | 128.75 (78.64) | -0.046 | 0.964 |
| PNI (Prognostic Nutritional Index) | 51.43 (8.39) | 52.75 (6.68) | -2.389 | 0.017 |
| SII (Systemic Immune Inflammation Index) | 497.89 (409.40) | 450.57 (376.97) | -1.750 | 0.080 |

**NLR = Absolute Neutrophil Count / Absolute Lymphocyte Count**

PLR = Absolute Platelet Count / Absolute Lymphocyte Count

PNI = Serum Albumin (g/L) + 5 × (Absolute Lymphocyte Count (10⁹/L))

SII = (Absolute Platelet Count × Absolute Neutrophil Count) / Absolute Lymphocyte Count

Table S4 Comparison of the basic characteristics between the training set and the validation set

| Characteristics | Training set  (n = 813) | Validation set  (n = 348) | χ²/Z | *P* |
| --- | --- | --- | --- | --- |
| Age (n, %) |  |  | 0.030 | 0.863 |
| ≤ 60 yr | 404 (49.7) | 171 (49.1) |  |  |
| > 60 yr | 409 (50.3) | 177 (50.9) |  |  |
| Gender (n, %) |  |  | 2.488 | 0.115 |
| Male | 646 (79.5) | 262 (75.3) |  |  |
| Female | 167 (20.5) | 86 (24.7) |  |  |
| Tumor location (n, %) |  |  | 0.120 | 0.942 |
| Upper third | 463 (56.9) | 202 (58.0) |  |  |
| Middle third | 146 (18.0) | 61 (17.8) |  |  |
| Lower third | 204 (25.1) | 85 (24.4) |  |  |
| Operation method (n, %) |  |  | 3.314 | 0.069 |
| Open | 563 (69.2) | 222 (63.8) |  |  |
| Laparoscopy | 250 (30.8) | 126 (36.2) |  |  |
| Type of resection (n, %) |  |  | 2.002 | 0.368 |
| Total gastrectomy | 398 (49.0) | 161 (46.3) |  |  |
| Proximal subtotal gastrectomy | 207 (25.5) | 84 (24.1) |  |  |
| Distal subtotal gastrectomy | 208 (25.6) | 103 (29.6) |  |  |
| Histological type (n, %) |  |  | 0.807 | 0.369 |
| Adenocarcinoma | 751 (92.4) | 316 (90.8) |  |  |
| Non-adenocarcinoma | 62 (7.6) | 32 (9.2) |  |  |
| Vascular invasion (n, %) |  |  | 2.189 | 0.139 |
| Yes | 379 (46.6) | 179 (51.4) |  |  |
| No | 434 (53.4) | 169 (48.6) |  |  |
| Neural invasion (n, %) |  |  | 2.267 | 0.132 |
| Yes | 379 (46.6) | 179 (51.4) |  |  |
| No | 434 (53.4) | 169 (48.6) |  |  |
| T stage (n, %) |  |  | 0.485 | 0.922 |
| T1 | 112 (13.8) | 34 (9.8) |  |  |
| T2 | 60 (7.4) | 30 (8.6) |  |  |
| T3 | 429 (52.8) | 198 (56.9) |  |  |
| T4 | 212 (26.1) | 186 (24.7) |  |  |
| N stage (n, %) |  |  | 1.281 | 0.734 |
| N0 | 233 (28.7) | 89 (25.6) |  |  |
| N1 | 122 (15.0) | 52 (14.9) |  |  |
| N2 | 172 (21.2) | 79 (22.7) |  |  |
| N3 | 286 (35.2) | 128 (36.8) |  |  |
| M stage (n, %) |  |  | 0.021 | 0.884 |
| M0 | 774 (95.2) | 332 (95.4) |  |  |
| M1 | 39 (4.8) | 16 (4.6) |  |  |
| TNM stage (n, %) |  |  | 1.179 | 0.758 |
| I | 132 (16.2) | 48 (13.8) |  |  |
| II | 190 (23.4) | 82 (23.6) |  |  |
| III | 453 (55.7) | 202 (58.0) |  |  |
| Ⅳ | 38 (4.7) | 16 (4.6) |  |  |
| ALB, (median, IQR) | 43.00 (5.0) | 42.90 (5.6) | -0.533 | 0.594 |
| CEA, (median, IQR) | 1.95 (2.44) | 2.00 (2.51) | -0.879 | 0.379 |
| ACR, (median, IQR) | 21.78 (27.72) | 21.42 (28.17) | -0.311 | 0.755 |
| ACR (n, %) |  |  | 0.354 | 0.552 |
| Low | 131 (16.1) | 61 (17.5) |  |  |
| High | 682 (83.9) | 287 (82.5) |  |  |
